# *Drosophila* as a Model System to Study Nonautonomous Mechanisms Affecting Tumour Growth and Cell Death

**DOI:** 10.1155/2018/7152962

**Published:** 2018-03-13

**Authors:** Jean-Philippe Parvy, Joseph A. Hodgson, Julia B. Cordero

**Affiliations:** ^1^CRUK Beatson Institute, Institute of Cancer Sciences, University of Glasgow, Garscube Estate, Switchback Road, Glasgow G61 1BD, UK; ^2^Wolfson Wohl Cancer Research Centre, Institute of Cancer Sciences, University of Glasgow, Garscube Estate, Switchback Road, Glasgow G61 1QH, UK

## Abstract

The study of cancer has represented a central focus in medical research for over a century. The great complexity and constant evolution of the pathology require the use of multiple research model systems and interdisciplinary approaches. This is necessary in order to achieve a comprehensive understanding into the mechanisms driving disease initiation and progression, to aid the development of appropriate therapies. In recent decades, the fruit fly* Drosophila melanogaster *and its associated powerful genetic tools have become a very attractive model system to study tumour-intrinsic and non-tumour-derived processes that mediate tumour development* in vivo*. In this review, we will summarize recent work on* Drosophila* as a model system to study cancer biology. We will focus on the interactions between tumours and their microenvironment, including extrinsic mechanisms affecting tumour growth and how tumours impact systemic host physiology.

## 1. Introduction

Despite being the most studied human disease, cancer remains a leading cause of mortality worldwide. Nearly 1 in 6 deaths in 2015 was attributable to cancer, according to the World Health Organization, with an increase of 70% of new cases projected within the next two decades [1]. The seemingly restricted success in controlling and reducing the devastating outcomes of this disease is due, to a great extent, to the high complexity and variable nature of the pathology. The current limited understanding of many aspects of cancer biology is partly imposed by limitations in conventional animal models of research.

The organismal implications and ultimate outcome of tumour burden in patients are undoubtedly determined by a combination of tumour-intrinsic mechanisms and interactions between tumours and proximal, as well as distal tissues [2–4]. While cancer research has classically focused on identifying tumour autonomous processes, there is a recent growing interest in understanding the nonautonomous mechanisms that control tumour progression [5]. Indeed, pioneering work dating back to the 19th century established the notion that distant tissues influence tumour growth and metastasis, when in 1896 Sir Beatson published a report on the treatment of inoperable cases of breast carcinomas through ovariectomy [2]. More recently, many molecular mechanisms have been identified highlighting the importance of the tumour microenvironment (TME) in cancer progression [5]. The crosstalk between tumour cells and their microenvironment often resembles normal physiological responses: for example, interactions between cancer cells and the immune system imitate various aspects of host-pathogen interaction [6]. In such a context, the body can detect cancer cells and react by mounting an immune response, to fight abnormal cell behaviours associated with the presence of a tumour. However, tumour cells appear to evolve to turn on new or divert existing physiological programs in order to evade the action of the immune system [6, 7]. The end result of such a power struggle between cancer cells and the surrounding tissues will ultimately determine the outcome of the tumour and its host. Targeting nontumoural tissues to counteract cancer growth is becoming a prime therapeutic strategy, which takes advantage of the higher genetic stability and lesser susceptibility of normal cells to escape drug treatments [8]. Hence, the discovery of novel non-tumour autonomous mechanisms to fight cancer progression is a promising area of research. However, the physiological complexity and limitations in the genetic accessibility of mammalian models systems render* in vivo* studies of non-tumour autonomous processes difficult to accomplish in conventional whole animal model systems.

*Drosophila melanogaster* remains the most powerful genetic model in research. During the last decades, the development of various tumour models, including leukaemia, neuroblastoma, glioblastoma, colorectal, and ovarian cancer, has made the fruit fly an attractive* in vivo* model system to decipher tumour intrinsic (i.e., tumour cell-autonomous) and extrinsic (i.e., non-tumour autonomous) molecular mechanisms mediating tumour growth and metastasis [9, 10]. Such studies have revealed astonishing conservation in the processes driving cancer development between flies and humans [10]. The ability to spatially and temporally regulate gene expression in tumour-bearing animals, as well as the low genetic redundancy, is particularly useful for the study of non-tumour autonomous mechanisms. Major advances in the understanding of these tumour-extrinsic mechanisms have been provided through the use of models based on loss of cell polarity, utilising mutants of the* scribble*-group of tumour suppressors genes (*scribble: scrib*,* lethal giant larvae: lgl*, and* disc large: dlg*), which encode key components of the basolateral polarity complex [11]. These mutations induce transformation of larval epithelial tissues, called imaginal discs, into “benign” neoplastic tumours. In this context, activation of proto-oncogenes, such as* Ras* or* Src, *drives tumour cell proliferation, spreading to distant tissues [12, 13]. During the years following the discovery of* scrib*-group genes as tumour suppressors in* Drosophila*, research has provided growing evidence that these models are directly relevant to human conditions. Indeed, scrib and dlg proteins are known targets of several oncogenic viruses, such as Human Papillomavirus, the main agent of cervical cancers. These viruses induce the degradation of the polarity complex proteins, comprising a key part of the process of malignant transformation in these conditions [11, 14, 15]. Loss of* scrib *has also been shown to work as a tumour suppressor in human breast, liver, skin, and lung cancers [16–19]. The loss of the human homolog of the Lgl protein has been involved in colorectal cancer [20] and hepatocarcinoma [21] and is associated with an increased risk of metastasis in endometrial cancer [22]. Moreover, similar to its* Drosophila* homolog,* scrib* also cooperates with the Ras oncogene to promote tumour cell invasion [12, 23].

Here, we discuss recent discoveries in* Drosophila *that have shed light into how extrinsic signals influence tumours, as well as mechanisms that mediate the systemic impact of tumours in the host. We focus on new findings highlighting the influence of immunity and metabolism in cancer progression and cancer-related disorders.

## 2. Cellular and Systemic Immunity Influence Tumour Growth and Cell Death

### 2.1. The Immune System: A Double-Edged Sword

Work in mammals has highlighted the immune system as a key component of the tumour microenvironment (TME), which plays a critical role in defining tumour outcome. While early studies on cancer patients support anticancer activity of the immune system [24], recent research has revealed that immunity can also promote tumour growth and metastasis [25]. However, deciphering the mechanisms of this dual immune function is a challenging task, mostly due to the complex cellular and molecular composition of the mammalian immune system [5]. For the past 15 years, the development of cancer models in* Drosophila* has allowed the discovery of molecular mechanisms mediating both pro- and antitumoural immunity. In contrast to mammals, which possess both innate and adaptive immunity,* Drosophila* only relies on innate immunity to fight against pathogens and tumours. Additionally, while mammals have numerous types of white blood cells, the cellular arm of* Drosophila* innate immunity includes only three main cells types—plasmatocytes, lamellocytes, and crystal cells—commonly called haemocytes. Only plasmatocytes have been currently reported to be associated with tumours [26]; however, a possible diversity within the haemocyte population bound to tumours cannot be excluded. Even if such macrophage-like cells were unable to infiltrate tumours as macrophages do, they could still produce a cocktail of mammalian-like cytokines leading to inflammation. While short-term inflammation can be beneficial to protect the host from challenges, such as those posed by pathogenic infection, chronic inflammation is associated with tumour initiation and metastasis in both* Drosophila* and mammals [27–29].

Tumour Necrosis Factor alpha (TNF-*α*) is a major proinflammatory cytokine produced within the TME, which was originally characterised for its ability to induce tumour death [30]. Consistently, TNF-*α*'s discovery led to great expectations for its use as a therapeutic target for cancer. However, further experiments have revealed a dual role for TNF-*α* as both an anti- and protumour factor [31]. The molecular bases of TNF-*α*'s antagonistic actions were poorly understood. However, recent research in* Drosophila *has highlighted some key molecular aspects underlying this dual action of the cytokine.* Drosophila* possesses a single TNF-*α* homolog called Eiger (Egr) [32, 33], whose role as an immune proinflammatory cytokine is conserved [34]. The importance of Egr in the TME has been highlighted in* Drosophila* tumour models through the use of mutants of the* scrib*-group of tumour suppressors genes. Egr expression is induced in tumours and tumour-associated immune cells [35, 36], much like mammalian TNF-*α*, which is detected in tumour cells as well as macrophages and T lymphocytes [31]. Given the focus of this review, we will only discuss the extrinsic role of Egr here. However, a tumour-intrinsic role of the cytokine has also been previously demonstrated [35, 37].

### 2.2. Cellular Arm of the Immune System and Associated Cytokines

**Figure 1:**
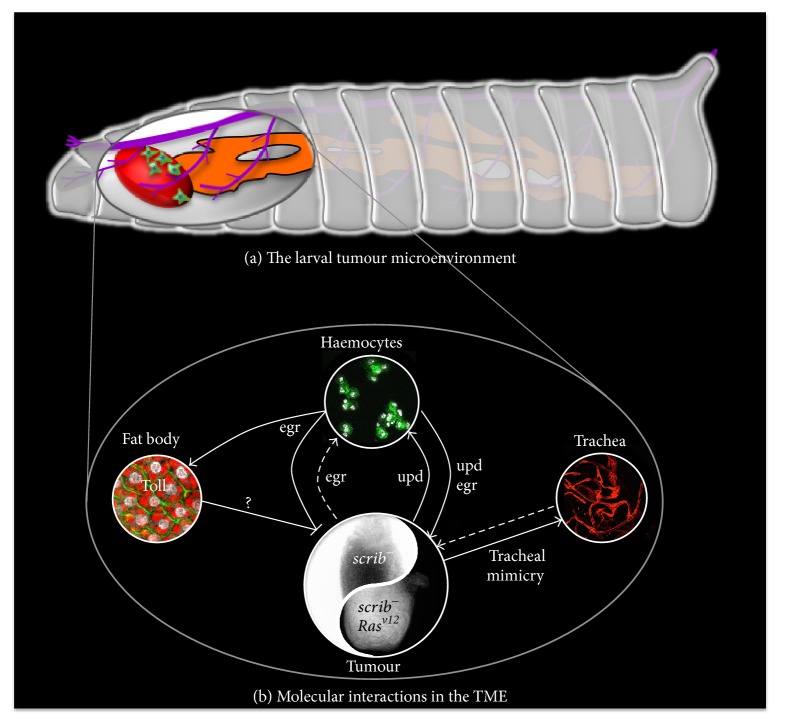
Immune interactions between larval tumours and their microenvironment (TME). (a) In* Drosophila* larvae, where tumours are generated in imaginal discs (tumour in red), the TME consists mostly of immune cells (in green), the fat body (in orange), and the trachea (in purple). (b) The molecular interactions within the TME are represented in this figure. Positive effects on growth and/or proliferation are highlighted by lines ending in arrowheads, while lines ending in bars show negative effects, mostly represented by increased cell death. Solid lines indicate demonstrated interactions and dashed lines potential ones. Both the immune cells and the tumour produce the fly TNF homolog Egr. It acts as a double-edge sword depending on the context of the tumour, represented as the Ying-Yang paradigm. Egr is antitumour in* scrib*-group mutant contexts, while being protumour and prometastatic when Ras^v12^ is present in the* scrib*-group mutant genetic background. The effect of tumour-derived Egr on immune cells is still an open question. Egr is required to activate the Toll pathway in the fat body, which subsequently promotes tumour cell death in combination with Egr itself, through an unknown signal (question mark). The interleukin homolog Upd3 produced by the tumour induces immune cells proliferation, while immune cell-derived Upd3 promotes tumour proliferation and invasion. While tumour can promote tracheogenesis through incorporation of tumour cells into the tracheal wall (tracheal mimicry), the effects of trachea on tumour growth and metastasis remain elusive.

Experimental evidence showed that immune cell-derived Egr has antitumoural activity. Patches of* scrib*,* lgl*, or* dlg* mutant cells generated in imaginal discs, delaminate, and are mostly removed from the epithelia through cell competition [12, 37–40]. However, in Egr mutant animals elimination of polarity deficient clones is abolished, and this effect can be recapitulated by knocking down* Egr* specifically within haemocytes, highlighting a conserved non-tumour autonomous anticancer function of Egr in* Drosophila* [35, 36] (Figure 1). Complementarily, loss of the TNF-*α* receptor Grindelwald (Grnd) in* scrib* mutant cells suppressed their removal from the epithelia [41]. In those cases, where a group of mutant cells is generated in a wild-type background, the elimination of mutant cells through cell competition relies on Egr-dependent JNK activation, which subsequently restricts cell proliferation and the survival of mutant cells [35, 38, 42]. This JNK-dependent toxic effect of TNF-*α* is conserved in mammals, as TNF-*α* induces cell death through TNFR1 and subsequent JNK signalling activation [29]. Recent discoveries of new molecules driving cell competition in* Drosophila*, including immune response proteins, may uncover new mechanisms involved in the elimination of cancer cells from a healthy tissue [43–45]. Egr has also been shown to exert antitumoural effects independently of cell competition. Full mutants animals for* scrib*-group genes, where neoplastic tumours develop from the whole imaginal disc, also show dependency from haemocyte-derived Egr to trigger JNK activation and tumour cell death [36, 46] (Figure 1). These studies highlight the importance of the TME and demonstrate a conserved antitumoural function of TNF-*α*-dependent inflammation in* Drosophila *models of cancer.

In contrast to the described antitumoural functions,* Drosophila *TNF-*α* can also exert protumoural effects. Evidence for such a role is provided by studies on tumours where* scrib-*complex mutations are associated with a constitutively active form of Ras (Ras^v12^). Ras is a conserved proto-oncogene mutated in many cancer types, with a 16% overall incidence rate in all analysed human tumours [47]. In* Drosophila*, clones of cells mutated for* scrib*-complex proteins and overexpressing Ras^v12^ fail to be eliminated by surrounding epithelial cells. Instead, they form neoplastic tumours that can invade distant tissues [12, 48]. While JNK is required for cell death in* scrib *mutant clones, cooperation with Ras^v12^ in these clones diverts the function of JNK pathway activation toward tumour cell proliferation and invasion [39, 49]. In this context, haemocyte-derived Egr has also been shown to promote JNK activation, as knockdown of Egr specifically in immune cells abolished JNK activation and restricted the ability of* scrib, *Ras^v12^ mutant cells to grow and invade. Strikingly, transplantation of Egr-wild-type immune cells could rescue the progression of* scrib, *Ras^v12^ tumours, as well as JNK activation, providing the final demonstration that* Drosophila *TNF shares protumour effects with its mammalian counterpart [36] (Figure 1). This is further supported by observations of high expression levels of Grnd in* scrib, *Ras^v12^ tumours and by data showing that* Grnd* knockdown in those tumours also disrupts their growth and invasive properties [41]. Interestingly, tumours display increased levels of ROS, which have been reported to promote haemocyte-dependent Egr secretion and subsequent JNK-induced proliferation in response to apoptosis, suggesting a protumoural feedback loop mechanism [50]. Further insights into the mechanisms mediating this protumorigenic role of Egr come from a recent demonstration that caspase-dependent ROS production in cancer cells is required for the recruitment of macrophages into *scrib*, Ras^v12^ tumours [51]. This work demonstrates that Ras^v12^-driven tumour progression requires the activation of Caspases, which function as tumour promoters. This mechanism is suggested to be one of the key mediators of the switch of Egr from an antitumour to a protumour cytokine by Ras. The protumoural function of TNF-*α* produced by immune cells is highly reminiscent to the one described in mammalian systems. In a mouse model of skin carcinogenesis where loss of TNF-*α* suppresses tumour formation [52], transplantation of B-cells from TNF-*α* competent mice is sufficient to restore tumour formation. However, this effect appears to be indirectly mediated through TNF-*α*-dependent regulation of T-cell number [53]. A more direct parallel between TNF-*α*-dependent antitumoural responses in flies and humans comes from work on Kras-dependent intrahepatic cholangiocarcinoma. In this context, TNF-*α* produced by Kuppfer cells (liver-specific myeloid cells) drives preneoplastic lesions through JNK signalling pathway activation [54].

The demonstration of antagonistic actions of TNF-*α* in* Drosophila* and mammalian tumours suggests that the successful use of antitumoural immunity as a cancer therapy may strongly depend on, and must take into consideration, the genetic composition of the tumour. This is further supported by data showing that not all neoplastic tumours are sensitive to Egr. The neoplastic growth induced upon knockdown of* avalanche (avl)*, a Syntaxin involved in the fusion of endocytic vesicles to the early endosome, is dependent on* Grnd* but escapes the need for* Egr* [41]. Interestingly,* avl* tumours produced high levels of Wingless (Wg) protein, which is a known target of JNK pathway activation and a key driver of compensatory proliferation, which is linked to cancer progression [55, 56]. It is therefore conceivable that the genetic properties and/or tissue location of a tumour dictate its sensitivity to different signalling pathways. A recent study in* Drosophila* showed that Wg dependent tumours proliferate independently of the TME and TNF-*α*/Grnd [57]. Similarly, tumours bearing combined loss of Ras^v12^ and hyperactivation of the nonreceptor tyrosine kinase Src, which also feature Wg overexpression [58], are largely insensitive to Egr loss (J.B.C. personal communication). High Wg activity could therefore be one of the factors rendering tumours insensitive to TNF-*α*. The expression of growth factors and activation of downstream signalling pathways in epithelial tissues in general and in* Drosophila* imaginal discs in particular are usually restricted to certain tissue locations [59, 60]. Recent work in* Drosophila* identified the presence of “tumour hot-spots.” Tumour hot-spots are defined as locations within tissues where neoplastic mutations are more likely to result in successful tumoural growths capable of invading normal tissues and it is a process involving differential activation of JAK/STAT signalling [61]. It is likely that additional spatially restricted factors, including graded morphogens, such as Wg, Decapentaplegic (Dpp), or Hedgehog (Hh), may influence “tumour hot-spots” and, therefore, the potential impact of TNF-*α* in this context.

A key phenotypic feature of* scrib*-group mutants is the loss of epithelial cell polarity. In tumours lacking* lgl*, knockdown of the JNK pathway rescues loss of cell polarity [62]. Loss of cell polarity is required for epithelial-mesenchymal transition (EMT), which drives tumour progression, including invasion [63, 64]. Given that Egr is a major driver of JNK pathway activation, the fly TNF-*α* may be a determinant in the loss of cell polarity in tissues carrying these neoplastic transformations. Indeed, Egr regulates asymmetric localisation of determinants of asymmetric division, Miranda and Prospero, in neuroblasts, supporting a role for Egr in cell polarity determination [65]. Interestingly, TNF*α*-dependent loss of cell polarity has been reported upon induction of chronic inflammation in the mouse intestine [66]. Likewise, a recent report shows that TNF*α*-dependent EMT increases lung cancer metastasis [67]. This possible relationship between TNF-*α* and cell polarity could also be the driving force for TNF-*α*'s protumour effect on Ras^v12^ expressing cells, as Ras hyperactivation facilitates the prosurvival function of JNK signalling.

The discovery of other immune-derived cytokines may have implications on their role in cancer progression through the TME. Haemocyte-derived Dpp, the fly homolog of Bone Morphogenetic Protein 2/4 (BMP2/4), a member of the Transforming Growth Factor beta (TGF-*β*) signalling family, can promote intestinal stem cell (ISC) proliferation in response to infection [68]. A similar effect has been reported in response to both septic and aseptic injuries for hemocyte-derived unpaired 2 and 3 (Upd 2/3), the* Drosophila* interleukin homologs that function as ligands of the JAK/STAT pathway [69]. Consistently, in* scrib* mutant larvae Upd 3 produced by the tumour induces JAK/STAT activation in the immune tissues (fat body and haemocytes), leading to a positive feedback loop that increases Upd 3 levels in haemocytes, which is required for JAK/STAT-induced proliferation of haemocyte and subsequent tumour suppression [26] (Figure 1). On the other hand, Upd3 can also impact JAK/STAT activation within* scrib*/Ras^v12^ tumour, where it cooperates with JNK to promote growth and metastasis [48] (Figure 1). A protumour effect of JAK/STAT signalling is also reported in fly leukaemia model, as its activation is sufficient to drive* Drosophila* blood cell neoplasia [70].

### 2.3. The Humoral Immune Response to Tumours

While the local immune response to tumours is receiving great interest for the design of new immunotherapies, the role of systemic immunity in mammals remains elusive. However, recent advances are highlighting the importance of systemic immunity to drive successful immunotherapy [71]. Pioneering work done in* Drosophila* has demonstrated a role of systemic or humoral innate immunity in the impairment of tumourigenesis. The main organ involved in humoral immunity in* Drosophila* is the fat body, which processes analogous functions to the mammalian liver and adipose tissues. Several conserved immune signalling pathways are activated in the fat body upon infection, including Toll, immune deficiency (Imd), and JAK/STAT signalling [72]. Activation of those pathways leads to the expression of downstream effectors (antimicrobial peptides, turandots, clotting factors, serine proteases, TEPs, serpins, and cytokines), which act by clearing the underlying infection and promoting recovery of infected tissues [73]. Interestingly, tumour-bearing animals show activation of the humoral immune response [46]. Unexpectedly, activation of the Toll signalling pathway in the fat body of tumour-bearing animals could be prevented by knocking-down the Toll ligand* Spaetzle (spz)* in haemocytes or by removing Egr from tumours, suggesting that Egr produced by the tumour promotes Spz production by haemocytes, which in turn activates the Toll pathway in the fat body [46]. Toll knockdown in the fat body leads to increased tumour size and decreased tumour cell death. Conversely, Toll overexpression is sufficient to induce tumour cell death and decrease tumour size, a process that requires haemocyte-derived Egr [46]. All together, evidence shows that TNF-*α*-dependent activation of systemic Toll signalling is an important component of a nonautonomous tumour suppressor program (Figure 1). The exact mechanisms of Toll activation, as well as the downstream effector(s) of the Toll pathway in tumour-bearing animals, remain elusive. Interestingly, downstream Toll targets expressed following infection include antimicrobial peptides (AMPs), which have been reported to exert antitumoural activity* in vitro* [74].

It is worth mentioning recent technical advances in flies that have provided new means to study the interactions between the tumour and the TME or more distant tissues. Tumour allografts have been a powerful technique to assess some physiological aspects of tumour growth and metastasis [57, 75–77], permitting independent genetic manipulation of tumours and non-tumour host tissues. Furthermore, it is likely that the use of new genetic tools that allow manipulation of gene expression independently from the widely used Gal4 system, such as the LexA/LexAop and QF/QS/QUAS systems [78, 79], will be extremely useful to study the influence of distant tissues on tumours. However, to this end the development of new fly lines is required, in order to establish these alternative gene-driving systems for use in large/unbiased screening of processes involved in tumourigenesis in* Drosophila*.

### 2.4. The Tracheal System and Its Role in Tumourigenesis

The vascular system of vertebrates is known to play a critical role in the tumour microenvironment, through interaction with the tumour and the immune system. Indeed, blood vessels deliver oxygen and nutrients, as well as immune cells, to all tissues. The fast-growing properties of cancers lead to the development of some hypoxic areas that are not vascularised. As a result, angiogenesis is required, in order to sustain the high demand for oxygen and nutrients necessary to ensure tumour growth. This therefore constitutes an attractive target for interfering with tumour development [80]. In* Drosophila,* oxygen is provided by the tracheal system that spreads throughout the animal, thus providing an analogous system to the vertebrate vasculature. Moreover, the* Drosophila* tracheal epithelium is also important in immunity, as it constitutes a physical barrier to the external milieu and is able to produce defence proteins [73]. Interestingly, a recent study showed that tracheogenesis occurs in the TME of hypoxic tumours in* Drosophila*. Strikingly, tumour cells undertake a trachea-specific developmental program and become incorporated into existing tracheal walls [81] (Figure 1). This data is reminiscent of the vascular mimicry process described in several mammalian cancer types, where tumour cells form functional blood vessel-like structures that can provide oxygen and nutrients to the tumour [82]. However, while tracheal derived Dpp is shown to influence ISC proliferation in the fly adult gut [83], the contribution of tracheogenesis to larval tumour growth and cell death and its possible contribution to antitumoural immunity remains an open question.

The studies described above highlight the importance of cellular and systemic immunity in shaping the tumour outcome. Critically, they reveal the existence of anti- and protumour mechanisms mediated by the immune system that are conserved between flies and humans and also uncover novel interactions between tumours and the immune system (Figure 1). However, even in a “simple” model system, interactions between tumour and immune cells are extremely complex. Future work in* Drosophila* will help to better understand how the global immune response shapes the TME, and how tumours are able to influence the antitumoural immune response via interactions with their microenvironment.

## 3. Interactions between Host Metabolism and Tumours

### 3.1. Tumours Impact Systemic Metabolism

**Figure 2:**
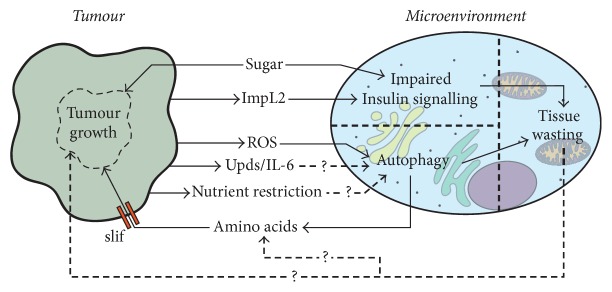
Metabolic interactions between tumours and their microenvironment (TME). Interactions between the tumour, the TME, and other environmental factors are represented in this figure. Solid arrows indicate demonstrated interactions, while dashed lines with question marks designate putative ones. Nonautonomous metabolic changes in the TME can affect both the TME and the tumour and are generated through various means. High levels of dietary sugar promote tumour growth and induce systemic insulin resistance in the TME. Tumours can also perturb TME insulin signalling by the secretion of an insulin-signalling antagonist, ImpL2. Autophagy in the TME promotes tumour growth through the recycling of amino acids from the TME into the tumour. Expression of the amino acid transporter* slif* in the tumour is necessary for this protumour effect. TME autophagy can be triggered by tumour-derived ROS and may also be driven by cytokine signalling or direct competition with the tumour for nutrients. Both, autophagy and impaired insulin signalling can contribute to tissue wasting and cancer cachexia. The causes of wasting in the TME and the effects of wasting in these tissues are an increasing research focus. However, the effects of TME wasting on the tumour remain an open question.

One of the striking effects of tumour burden is the alteration in host metabolism that occurs as a direct consequence of tumour development. The origins of the understanding that metabolism is altered in cancer patients can be traced back to the identification of glucose intolerance as the first systemic metabolic abnormality linked to the presence of a tumour [84]. This was followed by Warburg's discovery of the abnormal metabolism of glucose into lactate in tumours, which occurred even in the presence of oxygen [85]. Later discoveries have revealed a large panel of metabolic dysfunctions within tumours, which sustain further growth and proliferation of tumour cells. The high nutritional demand of tumours can influence nutrient availability in the TME, as demonstrated by recent work in mouse models showing that glucose restriction within the TME inhibits antitumour T-cell function [86, 87]. Furthermore, the high levels of hormone, peptides, and cytokine secretion observed during early tumour formation also affect metabolic pathways in distant tissues, leading to the hypothesis that tumours behave as “metabolic dictators” [88]. The biological complexity and limited genetic tools available in mammalian models, as well as the lack of physiological relevance of cell culture models to questions of interorgan communication, have largely hindered the investigation of altered host and tumour metabolism. As a model system,* Drosophila* has proven very relevant to the investigation of the links between tumour burden and altered systemic metabolism and the effects that this can have on both the tumour and host [89] (Figure 2).

### 3.2. The Effects of Diet on Tumour Burden

Obesity and type 2 diabetes are common comorbidities in modern society and are characterised by systemic insulin resistance and hyperglycaemia. These conditions are associated with an increased risk of developing cancer and are a risk factor for cancer mortality [90–94]. Insulin resistance can be modelled in* Drosophila* through the use of a high sugar diet, generating phenotypes that recapitulate the human condition [95]. In this context, small clones of noninvasive tumours cells transform into highly proliferative, metastatic tumours, due to the ability of these tumours to evade diet-induced systemic insulin resistance [58]. Tumours retain sensitivity to insulin signalling due to the overexpression of insulin receptor, as a result of elevated expression of Wg. This allows them to exploit the elevated levels of circulating glucose present in the context of the high sugar diet and peripheral tissue insulin resistance (Figure 2). It was later demonstrated [81] that activation of salt-inducible kinase in tumours from animals fed a high sugar diet functions to inhibit Hippo signalling, which facilitates the increase in Wg signalling that mediates the evasion of insulin resistance by these tumours. However, it is unclear whether nutrient availability has a universal impact on tumour growth, or whether any such dependency also relies on the genetic makeup of the tumour. An additional example of nutrient dependency can be identified in cells bearing a loss of function mutation in the tumour suppressor gene PTEN, which is commonly mutated across a broad range of cancers [96]. Under normal conditions, PTEN mutant clones in epithelial wing disc tissue show increased cell size but do not overgrow or disrupt tissue architecture. However, upon systemic nutrient restriction PTEN mutant cells display a proliferative advantage over wild-type cells, which is dependent on the function of the amino acid transporter* slimfast (slif)* [97]. Interestingly, overgrowth of PTEN mutant cells in the context of nutrient restriction was sufficient to induce systemic nonautonomous effects, decreasing the size of other tissues in the organism. PTEN mutant cells are suggested to outcompete distant wild-type cells for access to nutrients, as genetically driving growth in PTEN-competent peripheral tissues reduced the overgrowth observed in PTEN mutant cells [97]. Interestingly, the TOR pathway, a nutrient-dependent regulator of tissue growth, promotes the activity of Yki in wing discs [98], which is a known promoter of tumour growth [99–101]. This may therefore represent a possible mechanism by which increased nutrient availability promotes tumour growth in these* Drosophila* models. These findings demonstrate the drastic effect that the perturbation of host metabolism by extrinsic factors can have on tumour growth, how tumours exert systemic effects on distant tissues, and how the genetic properties of the tumour itself are critical in mediating this crosstalk.

Parallels can be drawn between the results observed in these* Drosophila *models and those found in vertebrates. Preexisting obesity and diabetes promoted tumour growth in a rat cancer model [94], while a study of over one million patients over 26 years identified diabetes as a predictor of both cancer development and cancer death [102].* Drosophila* cancer models involving diet and obesity are therefore particularly relevant to the human condition, as the protumour effects demonstrated in the contexts of these studies appear to be conserved in higher organisms, and the mediating factors are environmental influences that are very common in developed societies. The studies discussed here highlight new aspects of tumour physiology, suggesting that tumours are direct competitors to host tissues for nutrients and are frequently able to outcompete them for access to metabolic resources through various means (Figure 2). This induces nonautonomous metabolic effects in host tissues, which are likely to be beneficial to the tumour.

### 3.3. Non-Tumour Autonomous Autophagy and Tumour Growth

Macroautophagy is the process of bulk degradation of cytoplasmic components, facilitating the removal of defective organelles and the recycling and remobilising of cellular resources in times of stress [103]. While intratumour autophagy has been shown to act as a tumour suppressor, Ras^v12^ tumour cells in larval wing discs activate autophagy nonautonomously in the wild-type cells of the disc, demonstrating the ability of tumours to affect the TME in this manner [104]. This was further confirmed by another study reporting systemic non-cell-autonomous autophagy in animals bearing invasive neoplastic* scrib*/Ras^v12^ tumours [77]. Moreover, this study demonstrated that autophagic activity in tissues both local and distal to the tumour promoted tumour growth. Inhibition of autophagy in the local TME is sufficient to significantly inhibit tumour growth and invasion, an effect that is further enhanced when autophagy is also blocked in all peripheral tissues. These results directly demonstrate that non-cell-autonomous autophagy in local and distant nontumour tissues contributes to tumour growth and invasion [77]. These data are relevant to vertebrate models, as autophagy in pancreatic stellate cells has been demonstrated to promote tumour growth in a pancreatic cancer cell line implanted into mice [105].* Drosophila *studies have also suggested that microenvironmental autophagy fuels tumour growth through the mobilisation of nutrients from these local and peripheral nontumour tissues (Figure 2). It has been proposed that, in starvation conditions, autophagy induced by Desat1-dependent Myc activity may act in a non-cell-autonomous manner to promote tumour growth [106], while decreased amino acid transport, by the targeted knockdown of* slif* in the tumour, results in a dramatic loss of tumour growth [77]. In human cell culture models, microenvironmental autophagy has also been shown to metabolically support human pancreatic ductal adenocarcinoma in a non-cell-autonomous manner, through the provision of Alanine as a carbon source [107]. This shows that the data presented in these* Drosophila *studies is highly relevant to the vertebrate condition.

The tumour-derived factor(s) that drive the onset of microenvironmental autophagy are not yet fully defined; however, ROS signalling is an excellent candidate for further investigation (see Filomeni et al. [108] for a comprehensive review of ROS and autophagy). Starvation-induced autophagy is mediated by mitochondrially generated ROS, via the activation of the TOR pathway [109], while ROS are elevated in* scrib*/Ras^v12^ tumours, and the generation of mitochondrial ROS is sufficient to induce local autophagy in wing discs [77]. Manent et al. [104] provide evidence that ROS derived from tumour cells is sufficient to induce autophagy nonautonomously in the local microenvironment and that this also activates protumour JNK signalling in these cells. Altogether, these studies suggest that tumour-derived ROS might act as a convergent signal that triggers non-cell-autonomous microenvironmental autophagy and JNK signalling in the TME, both of which are protumour events (Figure 2). There is also some evidence in mouse models to support the idea that ROS may play an important role in TME autophagy. Fibroblasts that suffer oxidative stress induced by ROS and hypoxia in the TME undergo autophagy, which acts to degrade mitochondria. This alters the metabolism of these cells towards aerobic glycolysis, which, combined with autophagic degradation, is suggested to provide recycled nutrients from the TME to the tumour to fuel growth [110]. The transfer of energy between tumour and the TME in the form of metabolites is suggested to maintain the TME in a protumour setting [88]. Another recent work performed in cell culture and mouse models suggests that tumour-derived IL-6 may be a candidate for inducing autophagy in more tissues distal tissues from the tumour [111]. This work may represent an interesting novel target for the focus of research on the effects of peripheral tissue autophagy in* Drosophila* cancer models, as the expressions of IL-6-like Upd ligands are elevated in* Drosophila* neoplastic tumours [112]. There is little work exploring the potential interactions between ROS, autophagy, and Il-6 signalling in the context of the TME, and given the studies discussed here,* Drosophila *may represent a suitable model for further work into the interactions between these factors and their combined impact on the tumour and the TME. The importance of* Drosophila* studies on microenvironmental autophagy is reinforced by the apparent conservation of mechanisms in human patients and other vertebrate models systems. Further work in* Drosophila* is likely to be invaluable in improving our understanding of how metabolic changes in TME may affect tumours and shape tumour-host interactions.

### 3.4. Cancer-Associated Cachexia

One of the best-recognised outcomes of altered host metabolism in the context of tumour burden is the condition of cancer cachexia, a paraneoplastic syndrome that results in the dramatic loss of muscle and adipose tissue [113]. Cachexia is a highly multifactorial condition with numerous metabolic aberrations implicated in the onset of the condition, including perturbed insulin signalling, systemic hypercatabolism, inflammatory and immune responses, and deregulation of muscle homeostasis [114–117]. Cachexia is a highly deleterious condition, as it decreases patient tolerance to cancer therapies, negatively affects quality of life, and increases the risk of mortality, with up to 30% of cancer patient deaths occurring as a direct result of cachexia [118, 119]. Importantly, there is no clear therapeutic gold standard for the treatment of cachectic patients, in part due to the poorly understood aetiology of the condition. Cancer cachexia represents an extreme example of the effect a tumour can have on the host, as the presence of the tumour generates such a strong alteration of the host's metabolic state that it leads to the development of a novel pathology. There are unanswered questions about the systemic effects of cachexia beyond the direct effects of the wasting itself, including whether cachexia has a functional role that affects the tumour or other tissues.

Two independent models of cancer cachexia have shown the utility of* Drosophila* in this field of research [76, 120]. Both reports demonstrated that tumours secrete high levels of* imaginal morphogenesis protein-Late 2 (ImpL2)*, a secreted insulin-signalling antagonist that functions by direct binding to Dilp2 [121]. These studies also showed that tumour-bearing flies developed systemic insulin-resistance phenotypes in tissues distal from the tumour. This insulin resistance promoted tissue wasting, a process that is also likely to occur in human patients and other animal models [122–126] (Figure 2). RNAi knockdown of* ImpL2* in the tumour was sufficient to reduce the systemic insulin resistant phenotype and thus partially rescue the wasting phenotypes observed in peripheral tissues, without impacting the growth of the tumour [76, 120]. This work provides an excellent example of the use of* Drosophila* cancer models in the field of tumour-microenvironment interactions. Research into cachexia is an emergent field, and the identification of a tumour-derived factor that mediates a systemic effect on host tissue metabolism is an important example of the ability of* Drosophila *models to recapitulate and dissect complex phenotypes. Interestingly, autophagy is one of the main mechanisms of tissue degradation during cancer cachexia [111, 127, 128]. There are direct associations between whether tumours are cachectogenic and their ability to induce autophagy [111]. Together, these studies raise an interesting open question as to the functional nature of cachexia, namely, whether the process is not just deleterious to the host, but whether it is also beneficial to the tumour, due to the mobilisation of metabolites from muscle and adipose tissues. There are also questions as to whether tumour-inherent properties drive cachexia, and thus whether genetic factors can be established that mediate cachexia. Data from human patients suggest this may be the case, as pancreatic and gastric cancers have a much higher incidence rate of cachexia when compared to other tumour types [129, 130]. The* Drosophila* models discussed here represent a good opportunity to answer some of these important open questions.

## 4. Concluding Remarks

The studies discussed here demonstrate that* Drosophila* is a relevant model for studying cancer and its interactions with the TME, with many parallels to orthologous vertebrate conditions. Research utilising* Drosophila* as a model system has shown that immune and metabolic processes induced in a nonautonomous manner by the presence of the tumour are sufficient to feed back to the tumour and alter its characteristics. This can be shown well in the studies of microenvironmental autophagy, which is induced in the TME by the tumour, and serves to support tumour growth and metastasis [77, 104], and in the dual role of haemocyte-derived Egr, which can promote or suppress tumour growth depending on the tumour context [36, 46] (Figures 1 and 2).

Given the effects observed in response to the tumour there are likely to be interactions between the immune system and metabolism in this context. Indeed, nutrient restriction in larvae inhibits TOR signalling in the fat body, leading to increased levels of circulating Egr. Egr binds to insulin-producing cells in the brain and suppresses the production and secretion of progrowth Dilp2 and Dilp5 [131]. As previously discussed, Egr is also a mediator of tumour-induced immunity with context-dependent pro- or antitumour function [36, 41, 46]. There is therefore the potential for crosstalk between host tissues with tumour-derived metabolic derangement and immune pathways in* Drosophila. *Work in human cell culture and mouse models has demonstrated that tumours can alter host immunity via directly influencing immune cell metabolism. Lactic acid secreted by the tumour into the TME changes macrophages metabolism, polarising them towards a tumour-promoting state [132, 133]. These macrophages produce ARG1, a metabolic enzyme that generates polyamines (metabolites essential for cell division), which promote tumour growth in this context [134]. Another example is given by the direct competition for glucose between tumours and TME T cells, which is also sufficient to alter T-cell metabolism, suppressing antitumour responses and highlighting how the Warburg effect is used to escape the immune system [86, 87].

However, there is a lack of comprehensive understanding as to how different factors such as diet and metabolism, immune responses, and tumours interact and cooperate or synergise when presented together. This is often the case in the human condition, for example, in the case of a cancer patient with diabetes.* Drosophila* cancer models represent an excellent basis for the study of the roles these factors may play, both individually and combined together, and how nonautonomous signalling inputs might influence both tumour and host tissue responses. There are likely to be inevitable questions about the ability of simple* Drosophila* tumour models with one or two genetic drivers to fully recapitulate the complexity of tumour burden in higher animals, including human patients. However, the simplicity of these models is likely to prove advantageous when attempting to dissect the contributing roles of the multiple interacting factors that comprise tumour-TME interactions. There is also interesting work on the generation of* Drosophila* “avatars,” fly lines that can generate close homologs of tumours from specific patients, including the numerous genetic aberrations that drive a particular type of tumour in humans [135]. Such avatars may represent an excellent opportunity to test principles uncovered in more simple* Drosophila* cancer models, in order to investigate whether these discoveries still hold in a more complex tumour setting, including tumour-TME responses.

The mechanisms mediating the crosstalk between tumours and local and distal tissues are still being uncovered. Improving the understanding of the signalling pathways that may link together the complex interactions between host metabolism, immunity, and tumour growth is an essential aspect towards the unravelling of such crosstalk.* Drosophila* models represent an excellent platform for the continued investigation of these complex interactions, thanks to the multiple advantages of the model system. Low genetic redundancy, powerful genetic tools, and the possibility of tightly controlling not only the genetics of the tumour but also various aspects of the tumour micro- and macroenvironment render* Drosophila* a strong paradigm for further work into these complex interactions that impact human health and disease.
